# Not one, but many “publics”: public engagement with global development in France, Germany, Great Britain, and the United States

**DOI:** 10.1080/09614524.2020.1801594

**Published:** 2020-09-18

**Authors:** Jennifer Hudson, David Hudson, Paolo Morini, Harold Clarke, Marianne C. Stewart

**Keywords:** Aid – Aid effectiveness, Civil society – NGOs, Participation, Labour and livelihoods – Poverty reduction, Methods

## Abstract

Using new panel data from the Aid Attitudes Tracker (2013–18), this article draws on a set of 18 actions to map public engagement with global poverty in France, Germany, Great Britain and the United States. It introduces a new engagement segmentation comprised of five distinct groups – the totally disengaged, marginally engaged, informationally engaged, behaviourally engaged, and fully engaged. The data provide evidence of both aggregate and individual-level change in engagement over time but with an important distinction: respondents in less engaged groups are less likely to move out of these groups and tend to stay unengaged. Respondents in more engaged groups are more likely to move in and out of engagement.

## Introduction

Public engagement is a core feature of efforts by government, NGOs and charities to build support for aid and sustainable development. Engagement frequently comes in the form of awareness raising, communications, campaigns or development education programmes. At a foundational level, public engagement is thought to increase the effectiveness and legitimacy of government and NGO policies, and the ability of the public to hold development organisations accountable. Engaging the public is also felt to help anticipate, and respond to, public concerns about aid and development (e.g. waste, corruption, safeguarding, transparency). In OECD Development Assistance Committee (DAC) countries in particular, the public is thought to play a key role in achieving the Sustainable Development Goals (SDGs) and eliminating extreme global poverty: in supporting government spending on overseas aid and in taking private actions (i.e. donating, signing a petition or volunteering) to improve the lives of the world’s poorest (Darnton and Kirk 2011).

A key assumption underpinning efforts to build public support for development is the notion of an “engagement journey”, an organisations’ ability to increase individuals’ engagement with global poverty – from relatively low-cost actions to more intensive actions – through targeted communications and campaigns. At the organisational level, NGOs invest in efforts to build and monitor their supporters’ journeys; however, there is little systematic evidence on which actions, if any, the public take to engage with global poverty, or evidence of an engagement journey for the general public. We address these knowledge gaps here, providing the first comprehensive account of the actions the public take to engage with global poverty in France, Germany, Great Britain, and the United States. We address four questions: what kinds of actions do the public take; how does engagement change over time; what does a typical engagement journey look like; and what factors drive engagement with development organisations?

To address these questions, we draw on new panel data from the Aid Attitudes Tracker, 2013–18 (AAT). The AAT explores public attitudes and behavioural engagement with global poverty in four major donor countries: France, Germany, Britain, and the US. We introduce a new set of measures of actions the public take to engage with international development, comprising 18 different indicators. From these, we use latent class analysis to provide the first large-scale segmentation of public engagement with global poverty. We show how the five engagement groups – “Totally Disengaged”, “Marginally Engaged”, “Informationally Engaged”, “Behaviourally Engaged”, and “Fully Engaged” – move over time, illustrating the most common engagement journeys across the four countries, and explore the drivers of change that influence these.

We make three contributions to the literature on, and practice of, public engagement with global development. First, our segmentation shows not one but many “publics” and the largest groups – by far – are the Totally Disengaged and Marginally Engaged. Second, we show evidence of aggregate and individual-level change over time: engagement can rise and fall, but on balance net change is small. Third, and contrary to the received wisdom that individuals climb a “ladder of engagement”, we show patterns of engagement tend to remain fixed within a segment: respondents in less engaged groups are *less* likely to move out of these groups and tend to stay unengaged. These findings provide new evidence and challenges for development organisations who seek to build public engagement.

## Measuring engagement with global poverty

Previous research on public engagement has focused predominantly on understanding attitudes towards global poverty, and in particular, drivers of support for overseas aid (Scotto et al. 2017; Heinrich, Kobayashi, and Bryant 2016; Paxton and Knack 2011; vanHeerde and Hudson 2009). Less attention has been devoted to identifying the different ways in which the public directly take action on global poverty and how, if at all, these actions are related to one another. There are exceptions, notably studies on volunteering (Schech et al. 2015; Howard and Burns 2015; Devereux 2008) and donations (Bekkers and Wiepking 2011; Micklewright and Schnepf 2009; Sargeant 2009), but existing research has typically focused on a single action in isolation.^1^

There is good evidence that despite the global economic recession in 2008, at least in the UK, development organisations appeared to have “bucked the trend” of falling donations. Recent research on the UK sector from Banks and Brockington (2019) shows a 30% increase in the number of organisations between 2009 and 2015, and an overall increase in expenditure of 45% during the same period. “There is no evidence of a decline in income, including from the public who contributed 40% of the sector's income over that period, more than twice that of the next two largest sources combined” (Banks and Brockington 2019, 16). Yet despite robust income levels, there are growing concerns that the sector’s *transactional model* of public engagement is under strain: there are serious concerns over donor fatigue (Beswick et al. 2019), the dominance of outdated narratives (Seu and Orgad 2017), and concern over fewer donations and a drop in income resulting from the safeguarding crisis.

So called “cheap engagement” such as one-off donations or passively engaging with news and other social media, are the most common activities citizens take. Despite providing the cornerstone of public engagement for the past 30 years, the transactional model of engaging the public has been criticised. The arguments here are threefold. First, the model panders to the public’s preferences for convenient and arms-length forms of engagement. Second, it overlooks the structural causes of global poverty in suggesting that it can be addressed through a series of one-off donations. Third, by locking the public into low-cost/commitment forms of involvement, it only allows for incremental forms of political change which is at odds with the long-term horizons of fighting global poverty (Darnton and Kirk 2011).

Researchers have studied a range of indicators of engagement individually, for example, news consumption, donating, buying fair trade goods, buying charity branded products, volunteering time, lobbying MPs, becoming an activist, or joining a protest (Glennie, Straw and Wild 2012; House of Commons 2009), but have not examined a comprehensive list of actions, or considered the intensity and/or cost of individual actions. There have been significant efforts to develop such a list, that we build upon here. These include Bendapudi, Singh, and Bendapudi's (1996) framework for studying “helping behaviours” (which outlines three “types” of help: no help, token help and serious help) and Peloza and Hassay's (2007) typology of charity support behaviour which highlights six core actions involving high-involvement and low-involvement support actions. They also introduce the idea of low-involvement support working as a gateway to increased involvement and subsequent support with NGOs. We build on this logic of an engagement journey while expanding on the range of actions identified in the literature to outline 18 behavioural indicators to explore whether such a phenomenon exists.

## Findings

### Data and empirical approach

From 2013 to 2018, the Aid Attitudes Tracker (AAT) conducted surveys of public attitudes towards, and engagement with global poverty in France, Germany, Great Britain and the United States.^2^ The AAT is the first large, cross-national panel survey of how donor publics engage with global poverty (Clarke et al. 2013). Fieldwork was conducted using YouGov’s online panels with weighted nationally representative samples (n = c.6000–8000 respondents per country). The 10-wave panel was fielded twice-annually, starting in November 2013, with subsequent waves at six-month intervals. One of the distinctive features of AAT is its focus on behavioural engagement, including measurement of the 18 actions (Table 1), through which we study public engagement with global poverty.

**Table 1.** Aid Attitudes Tracker’s 18 engagement items.

Item	Which of the following have you done, if any, to become involved with international poverty and development as an issue?
1	Read, watched or listened to a news article about it, including offline and online
2	Discussed it with friends, family, or others in your community
3	Shared/forwarded an article or information about it including offline and online
4	Interacted with a community focused on the issue (e.g. join, follow, like/fan/friend, subscribed to a newsletter) including online and offline
5	Written on a blog, or commented on an article online
6	Used your voice to impact the issue (e.g. via social media, signing a petition, etc.)
7	Used online tools (such as Twitter, or Facebook) to share your opinions on the issue
8	Donated money to an organisation focused on the issue
9	Fundraised by asking for donations from others for a cause I am involved in (such as a charity, or trip)
10	Volunteered within the [country] for an organisation focused on the issue
11	Volunteered abroad for an organisation focused on the issue
12	Purchased products/services or boycotted products/services related to the issue
13	Voted specifically on the issue
14	Organised or helped to start/started a community focused on the issue, either online or offline
15	Organised or helped to set up an organisation focused on the issue
16	Contacted a Member of Parliament or other elected official in person or by phone call or letter about the issue
17	Contacted a Member of Parliament or other elected official online by clicking a petition or using Twitter, Facebook or other social media.
18	Participated in a march, rally, sit in, or other large event on the issue

Notes: Response options include: Have done in the last year; Have done, but not in the last year; Have never done; or Don't know. For the purpose of these analyses, we use only those who said they did the action in the past 12 months.

Source: Aid Attitudes Tracker, 2013–2018.

The items measure a wide range of activities through which respondents engage with global poverty, starting with more shallow engagement and getting progressively deeper. As such, they broadly reflect the idea of a “ladder” or “hierarchy of engagement” in terms of the time and commitment costs of taking each action. We expect that less-costly actions (e.g. watching news, donating) are more common than more-costly actions (e.g. going on a march or protest, volunteering). We assume that cost is measured not just financially, but also in terms of time and energy required.

**Figure 1.**
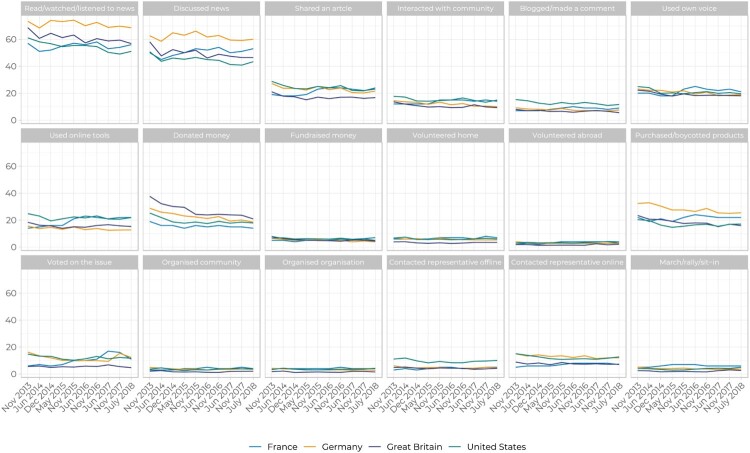
Percentage of respondents taking action in France, Germany, Great Britain and the United States, 2013–18.

The AAT panel highlights trends in public engagement across these 18 actions in our four countries from 2013 to 2018 (see Figure 1), from which we draw four key findings. First, and as expected, actions that are less costly are taken more frequently. Reading, watching, listening to news on global poverty (54%) and discussing it with friends and family (47%) are by far the most common ways of engaging across the four countries. More costly actions such as volunteering abroad (3%), setting up an organisation (3%), or going on a march/protest (4%) are far more infrequent. Second, while there is a great deal of consistency in the actions taken across countries, there are some important differences. Germans are far more likely to read, watch, listen or discuss news on global poverty, compared to the French or Americans. The British are far more likely to donate to development organisations and Americans are more likely to write to their representative on the issue.

Third, and importantly, despite significant efforts made by charities, NGOs and government agencies to increase public engagement with global poverty, Figure 1 shows that in most cases, levels of engagement remain unchanged, or worse, are in decline. Key among these is the percentage of respondents saying they have donated to an organisation in the past 12 months. Across all four countries have experienced a decline, and this is particularly acute in Germany and Great Britain. From 2013 to 2018 the percentage donating in Germany fell from 29% to 18%, and in Britain, from 37% to 18%. Fourth, the news is not all bad, particularly in France, where there have been measurable increases in discussing news, sharing articles, purchasing/boycotting products, and voting on the issue.

## Public engagement with global development

### Understanding audiences

The AAT data allow us to segment the public into different “engagement” groups across the four countries; our sample includes respondents that completed one or more of the 10 panel waves (France n = 24,836; Germany n = 14,848; Great Britain n = 22,450; US n = 16,944). Key to segmentation analyses is maximising similarities within and differences between groups. To estimate the segments, we use latent class analysis (LCA), which assumes that within the data are a set of unobserved, but existing sub-groups in the population (Collin and Lanza 2010). Similar to multiple correspondence analysis (MCA), LCA models assume that the latent or unobserved categorical variable – the engagement segments – account for the relations between the observed variables – the 18 actions. The claim we make is that there are distinctly different ways or patterns of engaging with global poverty issues: the classes are *qualitatively* different from one another, not just that people are more or less engaged (a quantitative measure).

The probability of being classified into one segment is based both on the type of action(s) taken, and their relative frequency to other actions. We specified the number of latent classes using model selection criteria (e.g. AIC, BIC) which discount model fit by the number of parameters estimated. The best-fit model produced five groups, each constituting a significantly different percentage of the total sample: Fully Engaged (2%), Behaviourally Engaged (6%), Informationally Engaged (13%), Marginally Engaged (33%), and Totally Disengaged (46%).

**Figure 2.**
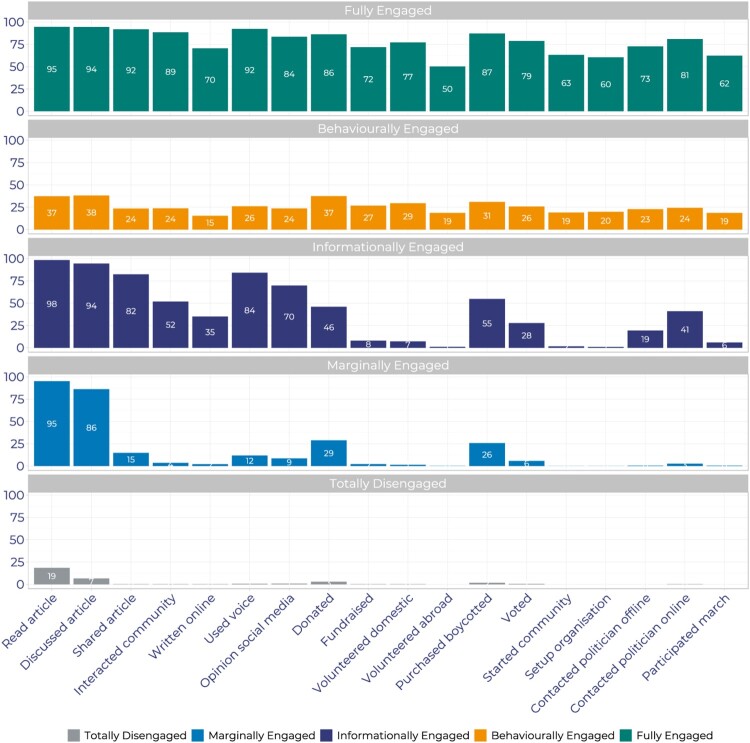
Percentage of respondents taking each action by segmentation group.

Figure 2 shows the percentage of respondents in the four countries that have taken each action by segmentation group. The first three groups are what we consider “engaged” audiences. The Fully Engaged (FE) and Behaviourally Engaged (BE) groups take all 18 actions, the key difference being the frequency in which they take action. The Informationally Engaged (IE) take actions relating to reading/watching or listening to news on the issue, but they also actively using their voice, for example interacting with a development organisation, signing a petition or writing/commenting on a blogpost. At the lower end of the engagement spectrum, the Marginally Engaged (ME) take typically low-cost (e.g. reading or watching news) and transactional actions (e.g. donating, purchasing or boycotting products). More than 9 in 10 (95%) of the ME say they read news on international development and global poverty and 86% discuss it with friends or family. While the ME do take other, stronger actions, they do so very infrequently. The TD take few, if any, actions, and the ones they do take are low cost. Thus, Figure 2 provides good prima facie evidence that the segmentation captures our concept of engagement, and these patterns hold when we disaggregate the data by country.

To get a better sense of differences underpinning the aggregate data, in Figure 2 we show the percentage of respondents in each of the engagement groups by country. In Table 2 we also show the key socio-demographic groups (age, women, high income, university degree) for each group.^3^ Several key similarities stand out, despite some important differences across countries. First, the median age of BE and FE groups is less than 40 years. This is significantly lower compared to the ME group where the median age is 50 + . Second, women are less likely to be in the more engaged groups: women are a majority of the two least-engaged groups (with the exception of the TD group in Britain). Third, university degrees are (on average) positively associated with higher levels of engagement across all four countries. Fourth, across the four countries the biggest difference lies in the relationship between higher income and engagement: in Germany and the US, respondents in higher income groups are more likely to be engaged, but this relationship is weaker in France and Britain.

**Table 2.** Engagement segments and their demographic characteristics in AAT countries.

	Totally disengaged	Marginally engaged	Informationally engaged	Behaviourally engaged	Fully engaged
**France**	** **	** **	** **	** **	** **
Group size (% of population)	28	37	16	13	5
Age	48	53	48	41	36
Women	52	57	51	46	39
High income	2	4	4	7	8
University	27	32	37	43	37
**Germany**					
Group size (% of population)	22	44	22	9	3
Age	47	53	53	39	37
Women	54	53	51	41	43
High income	5	11	11	15	17
University	14	25	26	34	34
**Britain**					
Group size (% of population)	32	35	17	13	3
Age	46	48	37	37	33
Women	45	53	52	46	43
High income	6	7	8	8	9
University	30	41	45	50	54
**US**					
Group size (% of population)	36	31	17	9	6
Age	47	51	55	34	39
Women	51	54	53	44	45
High income	19	24	29	25	32
University	20	28	42	25	44

Notes: Table shows the rounded percentage in each group. Median age is shown.

### Engagement journeys: aggregate and individual change over time

**Figure 3.**
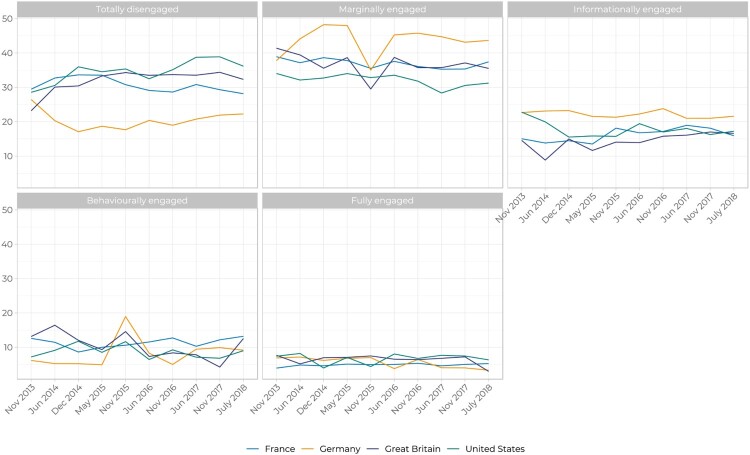
Aid Attitudes Tracker segmentation by country, 2013–18.

Having established the engagement groups, we now examine whether membership in these group is fixed, or whether and how people become more (or less) engaged over time. For development organisations, this is a question of whether people can be won or lost to the cause. Figure 3 shows the percentage of respondents in each country by level of engagement. There are a number of clear findings. First and most visibly, is that across all four countries the majority of the population are Totally Disengaged or Marginally Engaged. Second, Germany has the most engaged public. Third, while we do see evidence of over time change, it tends to be gradual and slow moving. The exception to this is the visible spike in November 2015 where there is a sharp aggregate shift from Marginally to Behaviourally Engaged in Germany and Great Britain. This spike aligns with the hundreds of thousands of Syrian refugees who arrived in Germany when the Dublin Procedure was suspended. This was accompanied with a large outpouring of concern and engagement among the population: many Germans volunteered to help welcome and house refugees or to provide blankets and shelter. But it is also noticeable that this trend appears to revert quickly to previous levels six months later.

**Figure 4.**
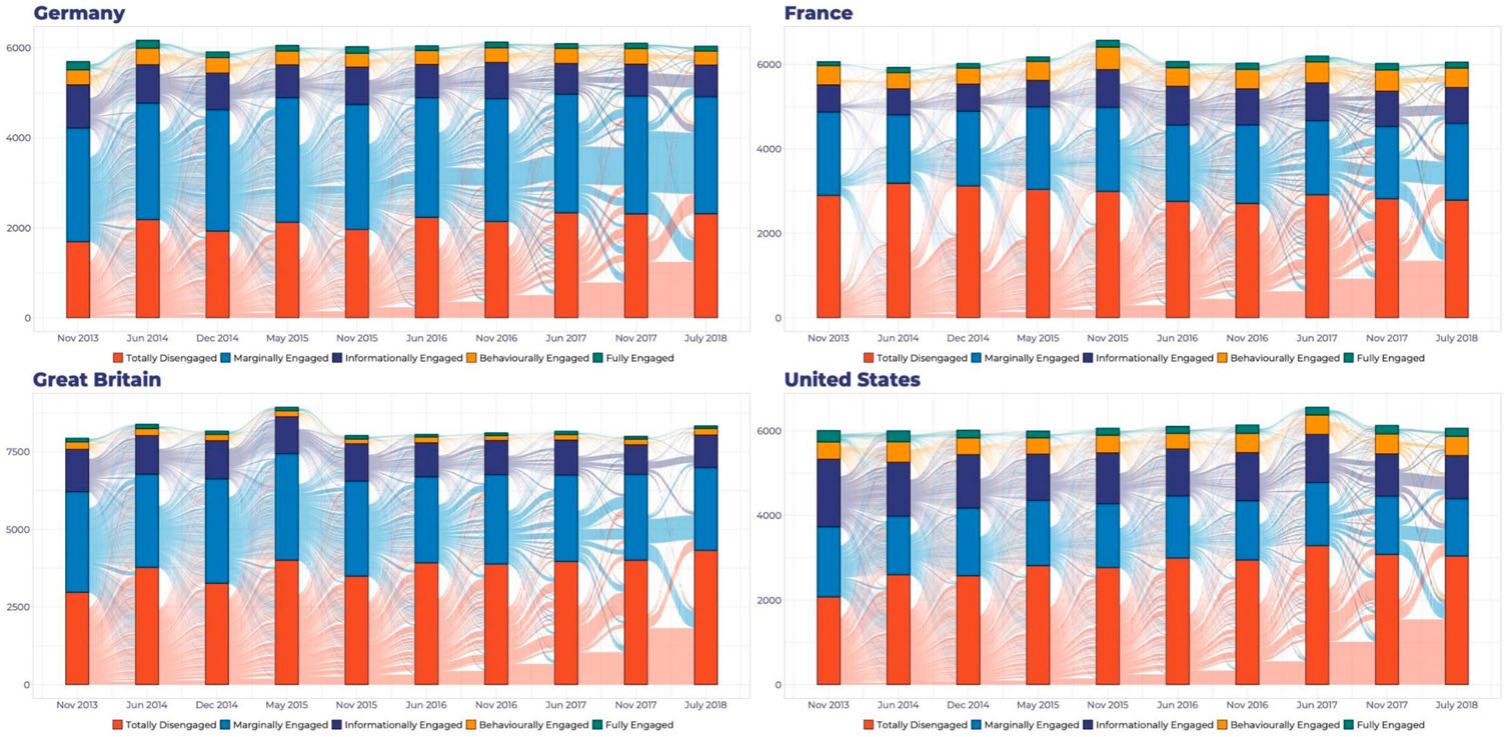
Individual wave–wave movement across segments.

The results in Figure 3 show that at the aggregate level there is movement within each engagement group, but wave on wave change is relatively small – and on balance – the trend lines can be characterised as stable. But small, aggregate-level change may mask more significant individual-level change. For example, while the overall percentage of ME group remains relatively constant, this could be the net result of entry and exit from other engagement groups. We can therefore deepen our understanding of engagement by looking at individual movement within these aggregate levels. Figure 4 uses Sankey plots to illustrate individual movements within and across the five groups over the 10 waves. Here too, we see clearly that the majority of respondents fall into the TD and ME segments. A second clear finding is the stability of these segments, especially within the lower two tiers of engagement. In other words, engagement levels are relatively *sticky*: respondents typically stay within their current segment over time. Third, contrary to the assumption of an upwardly mobile engagement journey, respondents move up *and* down the engagement ladder. Respondents who engage at high levels can also be lost.

In Table 3 we look at the average transition probabilities from one segment to another between any two waves. The initials along the rows show the segment transitioning from at time *t*, and the columns show the segment transitioning to at time *t+1*.^4^ The diagonal from the top left cell to the bottom right cell indicates the probability that a respondent will remain in the same segment from one wave to the next. The closer the number is to 1, the greater the probability a respondent will remain in the same segment.

**Table 3.** Average transition probabilities.

**France**	[-> TD]	[-> ME]	[-> IE]	[-> BE]	[-> FE]
[TD ->]	0.72	0.20	0.04	0.04	0.01
[ME ->]	0.35	0.50	0.10	0.04	0.01
[IE ->]	0.17	0.25	0.48	0.06	0.04
[BE ->]	0.27	0.18	0.11	0.39	0.05
[FE ->]	0.17	0.07	0.22	0.17	0.37
**Germany**	[-> TD]	[-> ME]	[-> IE]	[-> BE]	[-> FE]
[TD ->]	0.67	0.27	0.02	0.03	0.01
[ME ->]	0.22	0.67	0.08	0.02	0.00
[IE ->]	0.08	0.31	0.51	0.05	0.04
[BE ->]	0.23	0.23	0.13	0.35	0.05
[FE ->]	0.13	0.10	0.29	0.12	0.37
**Great Britain**	[-> TD]	[-> ME]	[-> IE]	[-> BE]	[-> FE]
[TD ->]	0.75	0.21	0.03	0.01	0.00
[ME ->]	0.31	0.59	0.09	0.02	0.00
[IE ->]	0.12	0.28	0.54	0.03	0.03
[BE ->]	0.30	0.26	0.18	0.22	0.05
[FE ->]	0.12	0.07	0.39	0.06	0.36
**United States**	[-> TD]	[-> ME]	[-> IE]	[-> BE]	[-> FE]
[TD ->]	0.74	0.16	0.05	0.04	0.01
[ME ->]	0.37	0.42	0.15	0.05	0.01
[IE ->]	0.16	0.21	0.50	0.07	0.05
[BE ->]	0.31	0.19	0.18	0.27	0.04
[FE ->]	0.16	0.06	0.30	0.09	0.38

We show that the TD and ME are the most stable or sticky segments, with an average transition rate over the 10 waves of 0.75 and 0.59 in Great Britain and 0.67 in Germany (both TD and ME). Across the four countries, the IE group tends to look less like the BE/FE groups and more like the TD/ME groups the in terms of stickiness: this is not surprising given that actions these groups take are relatively low-cost and homogeneous. What is striking is that the more engaged segments are less stable, meaning respondents are more likely to move in and out of them. Across the countries, there is less than a .40 probability of staying in the BE or FE groups, and in the case of the BE in Great Britain, a significantly lower probability (0.22). This suggests that it is harder to maintain the wider and more costly portfolio of actions the BE and FE do. The evidence here suggests that engagement journeys are not a pathway from low-cost to high-cost actions and engagement, or that there are effective “thresholds” of engagement that, once reached, are sustained.

### Sequence analysis – a look at typical “journeys”

**Figure 5.**
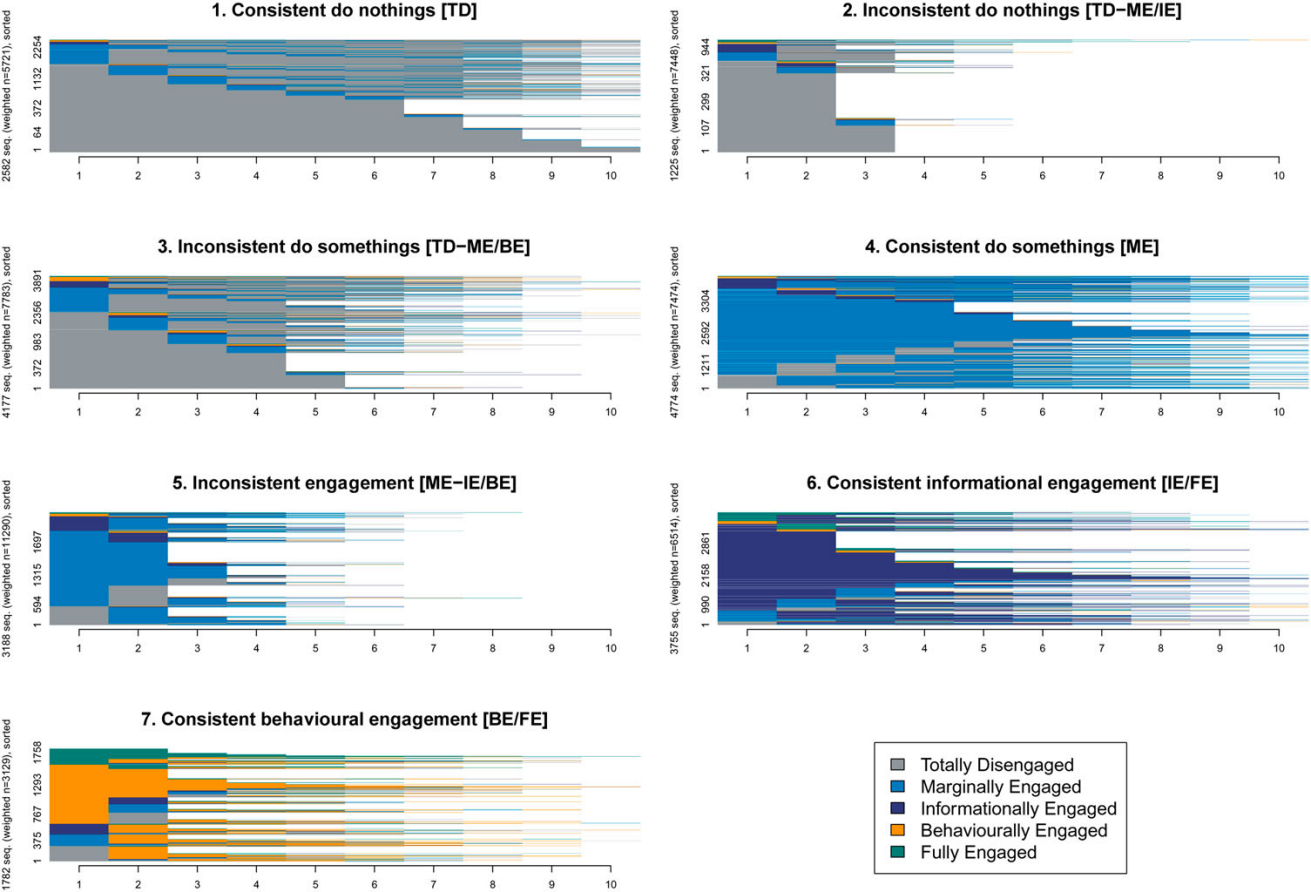
Sequence analysis: typical respondent journeys.

We extend our individual-level analysis by examining whether there are typical “journeys” or identifiable patterns in the journeys that respondents take. The assumption is that of a “linear” journey, from no or low-cost actions (Totally Disengaged) to higher-cost and frequency actions (Fully Engaged). We used the data to generate whole (engagement) sequences for each individual respondent who had completed more than one consecutive wave, resulting in 49,359 individual sequences (of which 21,483 were unique combinations of segments). We took every individuals’ engagement journey and grouped them into clusters that share similar journey patterns over time. We used optimal matching to calculate sequences that were more and less similar to one another by calculating the amount of additions, deletions, substitutions to transform one sequence into another (Abbott and Forrest 1986). Again, we use TraMineR for optimal matching (Studer and Ritschard 2016) and the distance matrix generated in a standard cluster analysis, identifying seven longitudinal clusters, shown in Figure 5.^5^ The x axis shows time, with all sequences left adjusted and the y axis stacks all of the individual journey sequences within each group.^6^

Figure 5 shows engagement journeys for all four countries combined and separate plots for individual countries tell a similar story. The plot shows the clusters of journeys, reading from left to right across rows and down, from the least to the most engaged. Clusters 1–3 show variations of the Totally Disengaged group. In cluster 1, *Consistent do nothings (TD*), respondents remain disengaged over the sequence, very rarely, if ever, moving out of this segment. In cluster 2, *Inconsistent do nothings (TD-ME/IE),* again the dominant state is to be TD, although we see some evidence of moving out of (and back into) TD. And in cluster 3, *Inconsistent do somethings (TD-ME/BE),* we see that again most journeys are typically that of being TD, but there is more frequent movement in and out of ME groups compared to cluster 2, and individuals in these groups can become Behaviourally Engaged.

We label cluster 4 *Consistent do somethings (ME),* as respondents in this journey are consistently Marginally Engaged, and only very rarely become TD. Respondents in cluster 5, *Inconsistent engagement (ME-IE/BE),* tend to flip-flop between ME and engaged groups. Finally, the last two clusters – cluster 6 *Consistent informational engagement (IE/FE)* and cluster 7 *Consistent behavioural engagement (BE/FE) –* reflect consistently engaged segments. Respondents in this cluster typically stay engaged, although they can move around within the IE, BE and FE groups.

The clustering of sequences allows us to infer a number of things. First, the overall story of stability is confirmed. Four out of the seven clusters tend to see respondents staying in the Totally Disengaged or Marginally Engaged segments: while there is some movement into a neighbouring segment, it is not sustained, and respondents tend to return to their equilibrium segment. Second, while there is more movement in clusters 6-7, they tend to stay engaged as opposed to become disengaged. Third, cluster 5, the *Inconsistently engaged*, is interesting for its relative size (23%) and mobility. These individuals are often in the ME group but do move in and out of one of the three engaged segments. Their journey is one of going back and forth as opposed to an escalating journey up the ladder of engagement.

### What drives changes in engagement?

Our final question concerns the factors driving changes in engagement. For this analysis, we focus on the ME group, the largest “state” as shown in the sequence analysis and a key group of interest to development NGOs and charities because of their perceived potential for moving up the engagement ladder. We model the impact of a defined set of predictors to explain why individuals move up (more engaged) or down (disengaged) from the Marginally Engaged group.

To do this, we pool all our respondents who are ME in waves t = 1, 3, 5, 7, 9, and check their levels of engagement in the following wave t+1 = 2, 4, 6, 8, 10. The differenced variable – *segdiff* – takes one of three values (1 = for those have gone from ME to TD, 0 = for those who remained ME, or 2 = for those who have become IE, BE or FE, grouped into a single category of “Engaged”) in a multinomial logistic regression model with fixed effects for waves and robust standard errors. The outcome variable, *segdiff,* is regressed on a set of predictors we observe at waves t = 2, 4, 6, 8, 10, measuring: support for *aid*; country and personal *economic evaluations*; non-utilitarian drivers of engagement, including *morality*, *social norms*, *trust* and *respect* for charities; *personal efficacy;* and *ideology* as measured by a left-right scale.^7^ We present the predicted probabilities as average marginal effects of the factor on being in one of the three engagement categories.

**Figure 6.**
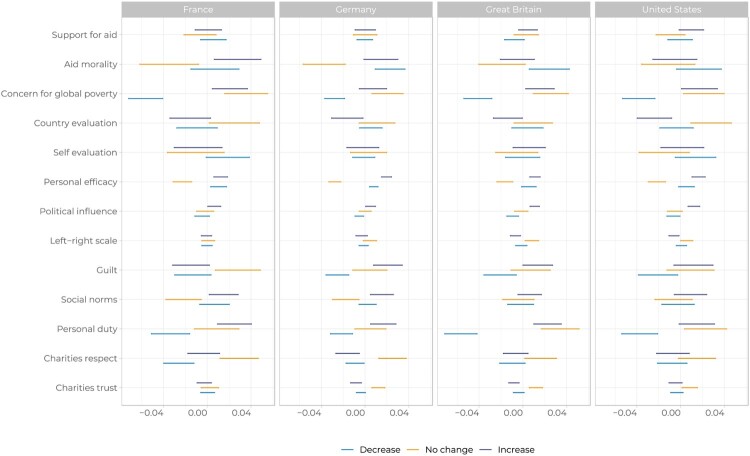
Predicted probabilities from the multinomial logistic regressions.

The results in Figure 6 show that the effects are not symmetrical or consistent across countries: what drives people down (from ME to TD) does not necessarily drive people up (from ME to Engaged). For example, the effect of *concern* for global poverty is negative and significant when moving from ME to TD, but we do not find an effect for concern for ME to IE, BE or FE (although positive). Being concerned about poverty, this shows, is not sufficient to drive up engagement levels: it keeps people in a Marginally Engaged state. We observe similar results when looking at *guilt*: people who feel guilty over not taking action are less likely to become TD, but the effect of *guilt* on becoming IE, BE or FE is inconsistent across countries. Just as importantly as the fact that there is no single direction in the engagement journey, there are also a different set of factors that drive engagement down or up.

The most consistent factor is *personal duty*: respondents with a higher sense of personal duty to tackle global poverty have a lower risk of disengaging, and a higher chance of moving to IE, BE or FE more than staying ME. We also find significant effects for respondents’ assessment of the country’s economic outlook: the more positive one’s view of the national economy, the less likely respondents are to move from IE, BE or FE to ME, but we find no effect for positive outlook on movement from ME to TD. We find no effect of *support for aid* or the evaluation of one’s own *economic status* on changing levels of engagement across countries.

Finally, we find an intriguing and counter-intuitive effect for *personal efficacy*, or the sense that respondents can personally make a difference to reduce poverty in poor countries. The positive coefficient for personal efficacy means that respondents are more likely to become TD and IE, BE or FE, relative to staying ME. In other words, increasing respondents’ sense that they can make a difference can tip them into becoming more and less engaged.

## Discussion and conclusions

This article has introduced an innovative new dataset – the Aid Attitudes Tracker – that offers novel insights into how the public in major donor countries take action to engage with global poverty and sustainable development. Using AAT data, we segment the French, German, British and US publics into one of five engagement groups, highlighting that engagement is best understood as not one, but many publics.

The motivating question for this research is: do we see evidence of an “engagement journey”? Do people start off from no or minimal activity, rise to the top, and stay there? While we hear such assumptions in common narratives, our data suggests that this is not the case. We show that there are changes in levels of engagement, but that people become more *and* less engaged at both the aggregate and individual level. This matters because it challenges the received wisdom, supported by evidence from within development organisations, that citizens can be taken on an engagement journey from relatively low-cost, low-frequency involvement to high-cost, high-frequency engagement. While some citizens do climb the engagement ladder, it is a very, very small percentage, and this movement does not represent the typical engagement journey across the four countries under study. The average engagement journey is from TD to ME, and to stay that way. These segments, our evidence shows, are sticky, in this respect. At higher levels of engagement (especially within the BE), segments become less sticky. Here individuals are likely to stay within an engaged segment (i.e. do not tend to fall into TD or ME segments), but are less likely to stay Fully Engaged. These findings are remarkably consistent across the four countries.

The Marginally Engaged are an important audience for development NGOs, not least in terms of their relative size. Generally speaking, they take some action(s) to engage with global poverty and do not have (strong) negative attitudes (e.g. they can be concerned about poverty in poor countries, are not overtly negative toward overseas aid, and feel a moral obligation to help people living in poor countries). They are an “eligible” audience to deepen engagement and will remain a target for organisations’ campaigns and communications. But it is important to recognise that moving the ME is not straightforward. The drivers analysis showed that with the exception of a sense of personal duty to address poverty in poor countries, there is an inconsistent set of drivers of engagement. Positive attitudes may prevent people from becoming less engaged, but may not be enough to increase engagement.

These findings have implications for development organisations. First, our findings challenge the notion of an engagement journey that dominates charitable organisations’ thinking, practice and offerings. If, as we have suggested here, donor publics don’t behave in the same way as “supporters”, this raises concerns that current practices to engage the public are not fit for purpose. Organisations’ communications and engagement opportunities may work well for supporters who have selected into organisations, but they may not work for a varied and diverse public. Second, the findings point to challenges for engaging each of the various segments or publics. If the least supportive groups – the Totally Disengaged and Marginally Engaged – are the hardest to move, and the engaged groups are less sticky, how should organisations respond? Finally, and looking ahead to 2030, we suggest the that SDGs present a good opportunity for reconceptualising development organisations’ public engagement strategies. This may require divesting from the traditional model of an engagement journey and investing in a more versatile (e.g. creative venues and touchpoints supported by new technology), broader (e.g. moving beyond extreme poverty to align with climate, global health and related issues) and bespoke sets of activities that fit a diverse public.

## Notes

1 The is a large literature on the drivers of charitable behaviour, including donations and volunteering. For example, Sargeant (1999) shows that charitable decisions (e.g. donating money, time, being loyal to a charity) are a function of inputs (appeals, information and asks), mediated by extrinsic determinants (age, gender, social class, income), intrinsic determinants (pity, empathy, social justice, self-esteem), perceptual reactions (how does the appeal fit with the self), and cognitive processing (based on past experiences and judgements). See also Bekkers and Wiepking 2006; Cheung and Chan 2000; Wilson 2000; Smith 1994.

2 The sample is Great Britain (England, Wales, Scotland) as it excludes respondents from Northern Ireland.

3 High income is a dummy coded 1 for individuals who earn more than £, € or $70,000 a year.

4 The transition rates are calculated using TraMineR (Gabadinho et al. 2011).

5 We used the K-Medoids algorithm which seeks to find the best partition of the data in a predefined number of groups by minimising the (weighted) sum of distance to the medoid of each group. The medoid is defined as the observation that minimise the sum of distance to the other observations of this group. We examined all the possible clustering solutions from 2 to 20 clusters and using substantive interpretation as well 10 statistical measures of the quality of the partitioning available in Studer’s Weighted Cluster package (Studer 2013).

6 For two different respondents their first wave could have been Wave 1 or Wave 7 depending on when they joined the panel – we assume no difference between the two. We also removed gaps in the data, so if an individual responded in Wave 7 and Wave 9 that would be shown in Figure 6 as Wave 1 and Wave 2.

7 Question wording for items in the multinomial regression model available on request.
